# Systemic 5-fluorouracil treatment causes a syndrome of delayed myelin destruction in the central nervous system

**DOI:** 10.1186/jbiol69

**Published:** 2008-04-22

**Authors:** Ruolan Han, Yin M Yang, Joerg Dietrich, Anne Luebke, Margot Mayer-Pröschel, Mark Noble

**Affiliations:** 1 Department of Biomedical Genetics and University of Rochester Stem Cell and Regenerative Medicine Institute, University of Rochester Medical Center, Elmwood Avenue, Rochester, NY 14642, USA; 2 Department of Neurology, Massachusetts General Hospital, Harvard Medical School, Fruit Street, Wang 835, Boston, MA 02114, USA; 3 Department of Neurobiology and Anatomy, University of Rochester Medical Center, Elmwood Avenue, Rochester, NY 14642, USA

## Abstract

**Background:**

Cancer treatment with a variety of chemotherapeutic agents often is associated with delayed adverse neurological consequences. Despite their clinical importance, almost nothing is known about the basis for such effects. It is not even known whether the occurrence of delayed adverse effects requires exposure to multiple chemotherapeutic agents, the presence of both chemotherapeutic agents and the body's own response to cancer, prolonged damage to the blood-brain barrier, inflammation or other such changes. Nor are there any animal models that could enable the study of this important problem.

**Results:**

We found that clinically relevant concentrations of 5-fluorouracil (5-FU; a widely used chemotherapeutic agent) were toxic for both central nervous system (CNS) progenitor cells and non-dividing oligodendrocytes *in vitro *and *in vivo*. Short-term systemic administration of 5-FU caused both acute CNS damage and a syndrome of progressively worsening delayed damage to myelinated tracts of the CNS associated with altered transcriptional regulation in oligodendrocytes and extensive myelin pathology. Functional analysis also provided the first demonstration of delayed effects of chemotherapy on the latency of impulse conduction in the auditory system, offering the possibility of non-invasive analysis of myelin damage associated with cancer treatment.

**Conclusions:**

Our studies demonstrate that systemic treatment with a single chemotherapeutic agent, 5-FU, is sufficient to cause a syndrome of delayed CNS damage and provide the first animal model of delayed damage to white-matter tracts of individuals treated with systemic chemotherapy. Unlike that caused by local irradiation, the degeneration caused by 5-FU treatment did not correlate with either chronic inflammation or extensive vascular damage and appears to represent a new class of delayed degenerative damage in the CNS.

## Background

Most treatments used to kill cancer cells also kill a diverse range of normal cell types, leading to a broad range of adverse side effects in multiple organ systems. In the hematopoietic system, the tissue in which such adverse effects have been most extensively studied, their detailed analysis has led to the discoveries that bone marrow transplants and cytokine therapies can improve the outcome of many forms of cancer treatment. In contrast, there has been no comparable level of analysis for most other organ systems compromised by cancer treatments.

One of the tissues for which adverse side effects of cancer treatment are clinically important is the central nervous system (CNS). Although it has long been appreciated that targeted irradiation of the CNS may be associated with neurological damage, it has become increasingly clear that systemic chemotherapy for non-CNS cancers also can have a wide range of undesirable effects. This has been perhaps most extensively studied in the context of breast cancer (for examples, see [1-13]). For example, it has been reported that 18% of all breast cancer patients receiving standard-dose chemotherapy show cognitive defects after treatment [9], with such problems reported in over 30% of patients examined two years after treatment with high-dose chemotherapy [10]; this is a greater than eightfold increase over the frequency of such changes in control patients. Adverse neurological sequelae include such complications as leukoencephalopathy, seizures and cerebral infarctions, as well as cognitive impairment [14-18]. Adverse neurological effects have been observed with almost all categories of chemotherapeutic agents [19-22], including antimetabolites (such as cytosine arabinoside (Ara-C) [23], 5-fluorouracil (5-FU) [24,25], methotrexate [26-28], DNA cross-linking agents (such as BCNU [29] and cisplatin [30]) and even anti-hormonal agents [31-37]. Given the large number of individuals treated for cancer, these adverse neurological changes easily may affect as many people as some of the more extensively studied neurological syndromes.

One of the most puzzling aspects of chemotherapy-induced damage to the CNS is the occurrence of toxicity reactions with a delayed onset. Although this has been particularly well documented in children exposed to both chemotherapy and cranial irradiation [15,38-47], delayed toxicity reactions also occur in individuals treated only with systemic chemotherapy. For example, white matter changes induced by high-dose chemotherapy for breast cancer, and detected in up to 70% of treated individuals, usually arise only several months after treatment is completed [48,49].

One widely used chemotherapeutic agent associated with both acute and delayed CNS toxicities is 5-FU. Acute CNS toxicities associated with systemically administered 5-FU (most frequently in combination with other chemotherapeutic agents) include a pancerebellar syndrome and subacute encephalopathy with severe cognitive dysfunction, such as confusion, disorientation, headache, lethargy and seizures. With high-dose treatment, as many as 40% of patients show severe neurological impairments that may progress to coma [50-52]. In addition, a delayed cerebral demyelinating syndrome reminiscent of multifocal leukoencephalopathy has been increasingly identified following treatment with drug regimens that include 5-FU, with diagnostic findings obtained by both magnetic resonance imaging (MRI) and analysis of tissue pathology [24,53-78].

Despite the existence of multiple clinical studies describing delayed CNS damage associated with systemic exposure to chemotherapy, almost nothing is known about the basis for these effects. For example, because of the multi-drug regimens most frequently used in cancer treatment, it is not even known whether delayed toxicities require exposure to multiple drugs. Nor is it known whether such delayed changes can be caused solely by exposure to chemotherapy or if they represent a combination of the response to chemotherapy and, for example, physiological changes caused by the body's reaction to the presence of a tumor. In addition, the roles of ongoing inflammation or damage to the vasculature in inducing such delayed CNS damage are wholly unknown. Moreover, the absence of animal models for the study of delayed damage makes progress in the biological analysis of this important problem difficult.

Here, we demonstrate that delayed CNS damage in mice is caused by short-term systemic treatment with 5-FU. Our experiments demonstrate that CNS progenitor cells and oligodendrocytes are vulnerable to clinically relevant concentrations of 5-FU *in vitro *and *in vivo*. More importantly, 5-FU exposure *in vivo *was followed by degenerative changes that were markedly worse than those observed shortly after completion of chemotherapy and that grew still worse with time. Systemic application of 5-FU *in vivo *(three injections interperitoneally (i.p.) over 5 days) was sufficient to induce delayed degeneration of CNS white-matter tracts. We observed this degeneration using functional, cytological and ultrastructural analysis and by altered expression of the transcriptional regulator Olig2, which is essential for generation of functional oligodendrocytes. The degeneration was not associated with either the prolonged inflammation or the extensive vascular damage to the CNS caused by local irradiation. This study provides the first animal model of delayed damage to white-matter tracts of individuals treated with systemic chemotherapy and suggests that this important clinical problem might represent a new class of damage, different from that induced by local CNS irradiation.

## Results

### Neural progenitor cells and oligodendrocytes are vulnerable to clinically relevant levels of 5-FU *in vitro*

We first examined the effects of exposure to clinically relevant concentrations of 5-FU *in vitro*, as in our previous studies on the chemotherapeutic agents cisplatin, BCNU (carmustine) and cytarabine [79]. To estimate clinically relevant concentrations, we used the following information: routinely used continuous intravenous infusions of 5-FU can result in steady-state plasma and cerebrospinal fluid (CSF) concentrations in the range 0.3–71.0 μM, and continuous pump infusions result in 3- to 25-fold higher levels of exposure [80]. High-dose (bolus) injections of 5-FU can even expose brain tissue to peak concentrations in the millimolar range [80,81], with tri-exponential elimination half-time values of 2, 12 and 124 minutes [82], and CSF elimination half-times can be greatly extended after localized application to brain tissue using slowly biodegradable polymer microspheres [83,84].

**Figure 1:**
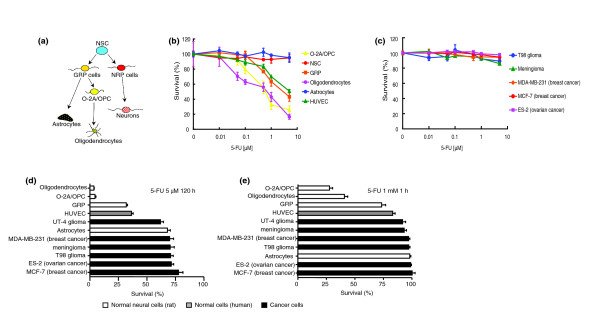
CNS progenitor cells are vulnerable to clinically relevant levels of 5-FU exposure. **(a) **A summary of the putative relationships between the different cell types under study (for discussion of this and alternative views on lineage relationships in the CNS, see [199,200]). Pluripotent neuroepithelial stem cells (NSC) give rise to glial restricted precursor (GRP) cells and neuron restricted precursor (NRP) cells. GRP cells in turn give rise to astrocytes and oligodendrocyte-type-2 astrocyte progenitor/oligodendrocyte precursor cells (O-2A/OPCs), the ancestors of oligodendrocytes. **(b,c) **Primary CNS cells (b) or various cancer cell lines (c) were grown on coverslips and exposed to 5-FU for 24 h before analysis of cell viability as described in Materials and methods. 5-FU concentrations were chosen on the basis of drug concentrations reached in humans after conventional 5-FU treatment. None of the tumor lines tested were sensitive to 5-FU treatment in this dose range, whereas O-2A/OPCs, oligodendrocytes, GRP cells and human umbilical vein endothelial cells (HUVECs) were sensitive. **(d,e) **Exposure conditions designed to mimic the exposure levels associated with long-term infusion (d) or high-dose bolus administration (e) yielded similar results, with vulnerability of O-2A/OPCs and non-dividing oligodendrocytes to 5-FU exceeding the vulnerability of rapidly dividing cancer cells. As shown in (b,d), the vulnerability of HUVECs also exceeds the vulnerability of cancer cells. Each experiment was carried out in quadruplicate and was repeated at least twice in independent experiments. Data represent mean of survival ± s.e.m, normalized to control values.

To identify potential targets of 5-FU toxicity, we first examined the effects of clinically relevant concentrations of 5-FU on purified populations of CNS stem cells, lineage-restricted progenitor cells and differentiated cell types. The cells examined were: neuroepithelial stem cells (NSCs) [85]; neuron-restricted precursor (NRP) cells [86]; glial-restricted precursor (GRP) cells [87,88]; and oligodendrocyte-type-2 astrocyte progenitor cells (O-2A/OPCs), the direct ancestors of oligodendrocytes [89], astrocytes and oligodendrocytes (the myelin-forming cells of the CNS). This is summarized in Figure 1a. For comparison, we also analyzed human umbilical vein endothelial cells (HUVECs) and cell lines from human breast cancer (MCF-7, MB-MDA-231), ovarian cancer (ES-2), meningioma and glioma (T98, UT-12, UT-4), and murine lymphoma (EL-4) and murine lymphocytic leukemia (L1210).

We found that progenitor cells and oligodendrocytes were vulnerable to clinically relevant levels of 5-FU. Exposure to 1 μM 5-FU for 24 hours (which is at the low end of the range of concentrations observed in the CSF of individuals treated with 5-FU by intravenous infusion [80]) caused a 55–70% reduction in viability of dividing O-2A/OPCs and also of non-dividing oligodendrocytes (Figure 1b). Exposure for 24 hours to 5 μM 5-FU killed about 80% of O-2A/OPCs and oligodendrocytes and more than 50% of GRP cells and HUVECs. Even at concentrations as low as 0.5 μM, 5-FU reduced the survival of O-2A/OPCs and oligodendrocytes by approximately 45%. Exposure to 5 μM 5-FU for 5 days killed almost all the oligodendrocytes (Figure 1c), and exposure to 1 mM 5-FU for just 1 hour reduced the number of viable oligodendrocytes by more than 55% (Figure 1d). In marked contrast, these doses of 5-FU had no effect on any of a variety of cancer cell lines, in agreement with previous studies on the breast cancer lines examined [90,91]. Thus, cell division was not sufficient to confer vulnerability to 5-FU, and a lack of division by oligodendrocytes was not sufficient to make them resistant.

Purified astrocytes and rapidly dividing NSCs were less vulnerable to 5-FU than progenitor cells and oligodendrocytes (Figure 1b–d), although even these populations showed some evidence of vulnerability when exposure time was extended to 120 hours (as is often associated with continuous intravenous infusion; Figure 1c). The relative resistance of NSCs to 5-FU (as compared with O-2A/OPCs, GRP cells and oligodendrocytes) demonstrates that, even in primary cell populations, cell division is not by itself sufficient to confer vulnerability to 5-FU.

We next investigated whether exposure to sublethal concentrations of 5-FU would disrupt normal progenitor cell function by suppressing cell division, as we have seen with BCNU, cisplatin and cytarabine [79]. Analysis of clonal growth in these experiments was used as it provides more detailed information on both cell division and progenitor cell differentiation than does analysis in mass culture. Progenitors, grown at cell densities that allow the study of single clonally derived families of cells (as in, for example, [92-94]), were exposed for 24 hours to 0.05 μM 5-FU (a concentration equivalent to less than 10% of that found in the CSF in standard-dose applications [81]), followed by 5 days of clonal growth.

**Figure 2:**
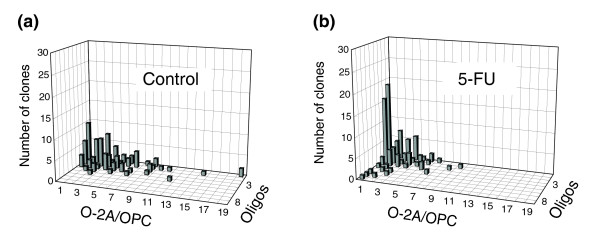
Sublethal doses of 5-FU inhibit division of O-2A/OPCs. Clonal analysis was used to study the effects of low-dose 5-FU (0.05 μM for 24 h) on the division and differentiation of freshly isolated progenitor cells. O-2A/OPCs were grown at clonal density and exposed one day after plating to **(a) **vehicle alone or **(b) **0.05 μM 5-FU for 24 h, doses that killed less than 5% of O-2A/OPCs in mass culture. The number of undifferentiated O-2A/OPCs and differentiated cells (oligodendrocytes) was determined in each individual clone from a total number of 100 clones in each condition by morphological examination and by immunostaining with A2B5 and anti-GalC antibodies (to label O-2A/OPCs and oligodendrocytes, respectively). Results are presented as three-dimensional graphs. The number of progenitors per clone is shown on the *x *(horizontal) axis, the number of oligodendrocytes on the *z *(orthogonal) axis and the number of clones with any particular composition on the *y *(vertical) axis. In 5-FU-treated cultures analyzed five days after initiating 5-FU exposure, there was an increase in the representation of small clones consisting wholly of oligodendrocytes and clones containing large numbers of oligodendrocytes, a reduction in the representation of large clones, a general shift of clone size towards smaller values, and a clear reduction in the total number of progenitor cells (see text for details). Experiments were performed in triplicate in at least two independent experiments.

Analysis of O-2A/OPC function at the clonal level indicated that transient exposure to 0.05 μM 5-FU caused suppression of O-2A/OPC division. Examination of the composition of 100 randomly selected clones showed that, at 5 days, the control cultures and the cultures exposed to 0.05 μM 5-FU contained similar numbers of oligodendrocytes (154 in control cultures (Figure 2a), and 175 in 5-FU cultures (Figure 2b)) but less than half as many O-2A/OPCs (336 in control cultures versus 151 in 5-FU cultures). There was a >85% reduction in the number of clones containing 8 or more progenitors (these clones comprised 13% of control cultures versus only 2% of 5-FU-treated cultures), along with a more general shift towards clones with fewer progenitors (Figure 2). There was also a greater than twofold increase in the number of clones consisting of just one or two oligodendrocytes and no progenitors. In control cultures, 16% of clones had such a composition, compared with 35% in cultures transiently exposed to 0.05 μM 5-FU. As clones were all initiated from single purified O-2A/OPCs, these results demonstrate that transient exposure of these progenitor cells to sublethal concentrations of 5-FU did not prevent the subsequent generation of oligodendrocytes, despite the adverse effects of even low-dose 5-FU on these cells (Figure 1b–d). As these cultures do not contain macrophages (which would ingest dead cells), cell death is easily observed by visual inspection and was found to be a relatively rare event, affecting ≤10% of total cells. Thus, it appears that the major cause of the lower cell numbers in 5-FU-treated cultures was a reduction in progenitor cell division, an interpretation consistent with the outcomes of the *in vivo *analyses discussed below.

### Systemic treatment with 5-FU causes increases in apoptosis and prolonged reductions in cell division in the adult CNS

**Figure 3:**
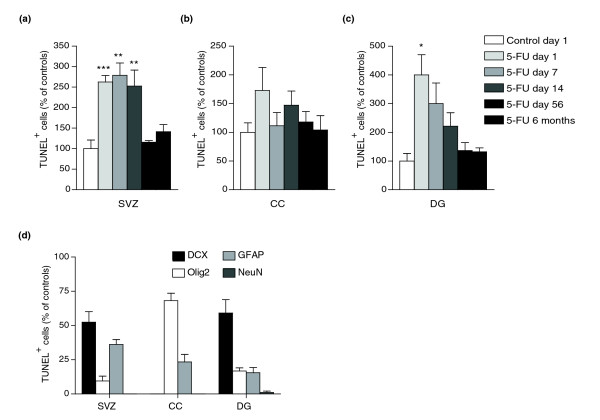
Systemic 5-FU treatment causes cell death in the adult CNS. Cell death was determined using the terminal deoxynucleotidyltransferase-mediated dUTP nick-end labeling (TUNEL) assay. The number of TUNEL^+ ^cells was analyzed in control animals (that received 0.9% NaCl i.p.) and 5-FU-treated animals and presented as percentage normalized values of controls at each time point. For ease of comparison, data presented in the figures show the control value (mean set at 100% of the day 1 value) and normalized values of 5-FU treatment groups at all time points. Each treatment group and the control group consisted of *n* = 5 animals at each time point. Figures show apoptosis in animals that received three bolus i.p. injections of 5-FU (40 mg kg^-1 ^on days -4, -2 and 0 leading up to the analysis, where day 1 of analysis equals 1 day after the last treatment with 5-FU). There was marked and prolonged increase of cell death in the 5-FU treatment group in **(a) **the lateral subventricular zone (SVZ), **(b) **the corpus callosum (CC) and **(c) **the dentate gyrus (DG) at 1, 7, 14 and 56 days and 6 months following treatment. Data are means ± s.e.m.; a two-way ANOVA test was performed on the original un-normalized data set to test the statistical significance of treatment effect and time effect. Bonferroni post-tests were performed to compare the 5-FU-treated group and the control group at each time point. The statistical significance of the Bonferroni post-tests is labeled in the graphs where applicable: ***p < 0.001; **p < 0.01; and *p < 0.05. Two-way ANOVA test results indicate that, in the SVZ, the treatment effect is extremely significant (*p *< 0.001), the time effect is very significant (*p *< 0.01); in the CC, the treatment effect is not quite significant (*p *= 0.06), the time effect is not significant (*p *= 0.74); in the DG, the treatment effect is extremely significant (*p *< 0.001), the time effect is significant (*p *< 0.05). The effect of the interaction between treatment and time is not significant for all three regions. **(d) **To determine the immediate cellular targets of 5-FU *in vivo*, we examined co-analysis of TUNEL labeling with antigen expression in animals sacrificed at day 1 after completion of 5-FU treatment. The majority of TUNEL^+ ^cells in the SVZ and DG were doublecortin (DCX)^+ ^ neuronal progenitors. Other TUNEL^+ ^cells in these two regions included GFAP^+ ^cells (which could be stem cells in the SVZ, or astrocytes in the DG) and Olig2^+ ^O-2A/OPCs. There was also a small contribution of NeuN^+^ mature neurons in the DG. In the CC, the majority of TUNEL^+ ^ cells were Olig2^+ ^(which, in this white matter tract, would be oligodendrocytes and O-2A/OPCs), with a small contribution of GFAP^+ ^astrocytes. Almost 100% of TUNEL^+ ^ cells were accounted for by known lineage markers. Each group consisted of *n* = 4 animals. Data are mean ± s.e.m.

*In vivo *treatment of mice with 5-FU (40 mg kg^-1^, 3 injections i.p. on days -4, -2 and 0 from the end of treatment; exposure determined as discussed in Materials and methods) caused significant induction of apoptosis in the multiple CNS regions examined (Figure 3a–c). For example, at day 1 after treatment, there was a 2.5-fold increase in apoptosis in the subventricular zone (SVZ) and a 4-fold increase in the dentate gyrus of the hippocampus (DG). The increased cell death persisted in the SVZ and DG for at least 14 days, but was at near normal values at 56 days and 6 months after treatment (Figure 3a,c). In the corpus callosum (CC) there was also a significant increase in apoptosis at day 1 to approximately 70% above control values (Figure 3b; *p *< 0.05).

Confocal microscopic analysis of immunolabeling and terminal deoxynucleotidyltransferase-mediated dUTP nick-end labeling (TUNEL) staining confirmed that the vulnerability of cells *in vivo *was similar to that observed *in vitro *(Figure 3d). In untreated animals, TUNEL^+^ cells (which are apoptotic cells) were very rare, but such cells were frequently found in the SVZ, DG and CC of animals receiving chemotherapy. In the SVZ and DG, the majority of TUNEL^+ ^cells observed after 5-FU treatment were double-cortin^+ ^(DCX^+^) neuronal progenitors [95], followed by GFAP^+ ^cells (a subset of which may be stem cells in the SVZ [96]). The SVZ also contained a smaller number of TUNEL^+^ Olig2^+^cells, which could be ancestors of oligodendrocytes [97,98]. In the DG, there was also a very small amount of NeuN^+ ^mature neurons that were TUNEL^+^. In the CC, approximately 70% of the TUNEL^+ ^cells were Olig2^+^, and thus would be either oligodendrocyte progenitors or oligodendrocytes. Most of the remaining TUNEL^+ ^cells in the CC were GFAP^+^, which in this tissue would mean they are astrocytes. The specificity of TUNEL labeling is demonstrated by representative images of TUNEL^+ ^cells that were DCX^+^, Olig2^+ ^or GFAP^+ ^(Additional data file 1).

**Figure 4:**
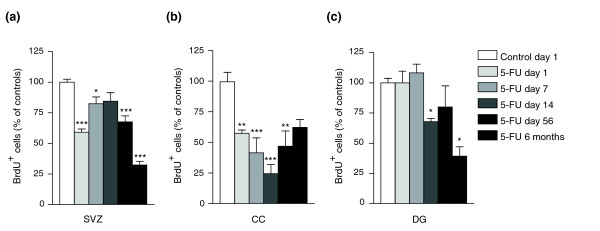
Systemic 5-FU exposure causes prolonged suppression of proliferation in the adult CNS. Animals were treated as described in Figure 3. The number of BrdU^+ ^cells was analyzed in control animals and 5-FU-treated animals and presented as percentage normalized values of controls at each time point. For ease of comparison, data presented in the figures show the control value (mean set at 100%) of day 1 and normalized values of 5-FU treatment groups at all time points. Each group consisted of *n* = 5; a two-way ANOVA test was performed on the original un-normalized data set to test the statistical significance of treatment effect and time effect. Bonferroni post-tests were performed to compare the 5-FU-treated group and the control group at each time point. The statistical significance of the Bonferroni post-tests is labeled in the graphs where applicable: ***p < 0.001; **p < 0.01; or *p < 0.05. Two-way ANOVA test results indicate that: **(a)** in the SVZ, both the treatment effect and time effect are extremely significant (*p *< 0.001), and the interaction of treatment and time is very significant (*p *< 0.01); **(b)** in the CC, both the treatment effect and time effect are extremely significant (*p *< 0.001), and the interaction of treatment and time is very significant (*p *< 0.01); and **(c)** in the DG, the treatment effect is very significant (*p *< 0.01), the time effect is extremely significant (*p *< 0.001), and the effect of the interaction between treatment and time is not significant.

Analysis of cell division (as detected by incorporation of 5-bromo-2-deoxyuridine (BrdU)) revealed that 5-FU caused long-lasting suppression of proliferation in the SVZ and the DG [99,100] (in which such proliferation is thought to be a critical component of normal tissue function) as well as in the CC (Figure 4a–c). Exposure to 5-FU caused reductions of cell proliferation in all three regions. In contrast with the return of levels of cell death to control levels (at least as detected by TUNEL staining), cell division was suppressed for long periods of time following completion of 5-FU treatment.

In the SVZ, 5-FU exposure was associated with a 40.9 ± 2.6% decrease in numbers of BrdU^+ ^cells on day 1, with a transient re-population of BrdU^+ ^cells at days 7 and 14, followed by a subsequent decrease in animals examined at day 56 and 6 months after completion of treatment. It was striking that the most significant inhibition of DNA synthesis in the SVZ was seen at 6 months post-treatment, when there was a 67.7 ± 3.0% decrease in the number of BrdU^+ ^cells compared with control animals (Figure 4a). In the DG, suppression of DNA synthesis started on day 14 after treatment, and the greatest inhibition (60.7 ± 7.8%) was also seen at 6 months (Figure 4c). In the CC, in contrast, cell proliferation was significantly suppressed at all time points examined (Figure 4b).

To determine whether exposure to 5-FU preferentially reduced DNA synthesis in any particular cell population(s) *in vivo*, we combined BrdU labeling with cell-type-specific antibodies and analyzed individual BrdU^+^ cells by confocal microscopy (see Materials and methods). We analyzed the CNS of animals sacrificed 1 day and 56 days after the completion of 5-FU treatment in order to examine the acute and long-term effects of treatment.

**Figure 5:**
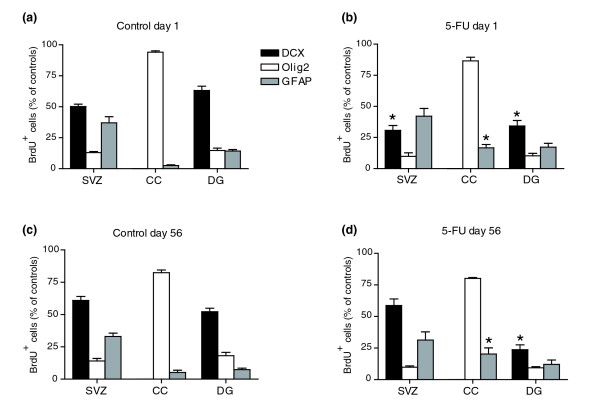
Cell-type analyses of BrdU^+ ^cells in control and 5-FU-treated animals at early and late time points after completion of treatment. Co-analysis of BrdU incorporation with antigen expression was conducted as described in Materials and methods. Both control and 5-FU-treated groups were analyzed at **(a,b)** day 1 and **(c,d)** day 56 to evaluate the immediate and long-term effects of 5-FU treatment. Results indicate that division of both DCX^+ ^neuronal progenitors and Olig2^+^oligodendrocyte precursors was reduced by systemic exposure to 5-FU. In the CC, the reduction in apparent division of Olig2^+ ^cells was proportionate to the overall reduction in all BrdU^+ ^cells. In the SVZ, there was an enhanced reduction of DCX^+ ^cells from among the BrdU^+^population at day 1 but not at day 56. In the DG, there was an enhanced reduction in the dividing DCX^+ ^population at both day 1 and day 56. In addition, the proportion of GFAP^+ ^cells in the CC was increased among the BrdU^+ ^population at both time points examined. Data are mean ± s.e.m; *p < 0.05, in comparisons with control animals (confidence interval = 95%, by unpaired, two-tailed Student's *t*-test).

We found that neuronal precursors and oligodendrocyte precursors were both affected *in vivo*. In the CC, where there was a 42.6 ± 2.7% reduction in the number of BrdU^+ ^cells in tissue sections from animals sacrificed 1 day after the completion of treatment (Figure 4b), the proportion of BrdU^+ ^cells that were Olig2^+ ^was similar between controls and treated animals (Figure 5a,b). This result also held true at day 56, when the proportion of Olig2^+ ^cells among the BrdU^+^population was unchanged in untreated and treated animals, despite a continued 53.2 ± 12.4% reduction in the total number of BrdU^+^cells observed (Figure 5c,d). As >90% of the BrdU^+ ^cells in the CC were Olig2^+^, these results indicate that the reduction in DNA synthesis observed in this tissue predominantly affected O-2A/OPCs [97,98,101].

In contrast with effects on putative O-2A/OPCs, there was a somewhat enhanced loss of DCX^+ ^cells (which would have been neuronal progenitors [95]) from among the BrdU^+ ^population in both the SVZ and the DG (Figure 5a–d). In the SVZ, at 1 day after treatment, there was a disproportionate and significant reduction in the percentage of DCX^+ ^ BrdU^+ ^cells, which represented 50.2 ± 1.9% of the cells incorporating BrdU in control animals and only 30.7 ± 3.9% in animals treated with three injections of 5-FU (*p *< 0.01). At day 56 the proportion of BrdU^+ ^cells that were DCX^+ ^was not different between controls and treated animals, although the total number of BrdU^+ ^cells in the SVZ of treated animals continued to be significantly lower than that of the control group (only 67.7 ± 4.9% compared with control animals at the same time point; *p *< 0.01). In contrast, in the DG, a reduction in the number of DCX^+ ^cells was also seen, both at day 1 (with DCX^+ ^cells comprising only 34.3 ± 4.4% of the BrdU^+ ^population in 5-FU-treated mice compared with 63.2 ± 3.4% in the control mice; *p *< 0.01), and at day 56 (23.7 ± 3.9% in 5-FU-treated mice versus 52.2 ± 2.8% in the control mice; *p *< 0.01). In the CC, exposure to 5-FU was also associated with a small increase in the proportion of GFAP^+ ^cells among the BrdU-incorporating populations at both day 1 and day 56, although such cells continued to represent a minority of the BrdU^+ ^cells in this tissue. In addition, BrdU^+ ^cells that were not labeled with any of the cell-type-specific antibodies used in these studies were more prominent in treated animals than in controls at day 1 (but not at day 56) in the SVZ and were found in the DG at both time points (data not shown). The DG was the only tissue in which these unlabeled cells made up >10% of the entire BrdU^+ ^population. Such cells represented about 40% and 50% of all BrdU-labeled cells in 5-FU-treated animals at days 1 and 56, respectively, compared with about 2% and 20%, respectively, of all BrdU-labeled cells in control animals.

### Analysis of auditory function in 5-FU-treated animals suggests delayed disruption of myelination

To determine whether the exposure of experimental animals to 5-FU was associated with functional impairment, we investigated hearing function in treated animals at various time points after treatment. Damage to the auditory system is a well known correlate of treatments with cisplatin [102,103]. This damage is associated with death of cochlear outer hair cells, increases in the auditory brainstem response (ABR) thresholds and decreases in transient evoked otoacoustic emissions (TEOAE) and distortion product otoacoustic emissions (DPOAE), all of which are indicators of compromised cochlear function.

We examined the DPOAE as an indicator of cochlear function and ABRs to provide information on changes in conduction velocity from the ear to the brain, an indicator of myelination status. Different peaks (called P1, P2, and so on) in the ABR response are thought to correspond to different steps in the transmission of information, and prior analysis of ABR inter-peak latencies shows that loss of myelin (as in, for example, CNS myelin-deficient mouse models [104,105]) causes increases in specific ABR inter-peak latencies (P2-P1 and P3-P1). Such measurements have been used by several investigators to study myelination-associated problems in impulse conduction in children with iron deficiency [106-109].

Our analysis of auditory function in 5-FU-treated animals revealed what seems to be a previously unrecognized consequence of chemotherapy exposure: increased latencies of impulse transmission. Consistent with the absence from the literature of reported deficits in cochlear function associated with 5-FU administration, DPOAEs in treated animals were not significantly different from those in untreated animals. In contrast, treated animals showed a progressive alteration in ABRs when inter-peak latencies were examined at days 1, 7, 14 and 56 after completion of treatment and compared with baseline measurements of each individual 1 day before 5-FU application.

**Figure 6:**
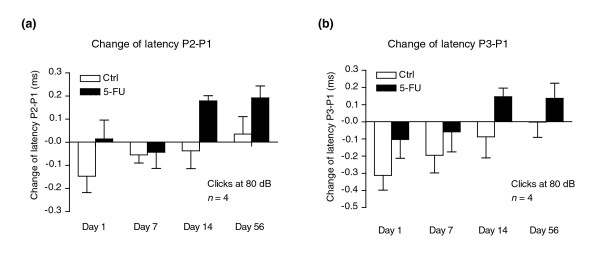
Systemic 5-FU treatment caused delayed increases in auditory brainstem response (ABR) inter-peak latencies P2-P1 and P3-P1. Baseline ABR hearing tests were performed on each animal one day before initiation of treatment with 5-FU (as for Figure 4). After treatment ended, follow-up ABR tests were conducted on each animal at various points during a time course of 56 days. Control and treatment groups both consisted of *n* = 4 animals. ABR latencies were analyzed for each individual at each time point, and change of latency was calculated as L_t _– L_0 _(L_t_, latency values at day 1, day 7, day 14, or day 56 post treatment; L_0_, baseline latency values 1 day before treatment initiation). **(a)** The change of inter-peak P2-P1 latency values; **(b)** the change of inter-peak P3-P1 latency values. At the later time points day 14 and day 56, both P2-P1 and P3-P1 inter-peak latency values of 5-FU-treated animals show average increases of more than 0.13 ms, whereas the same inter-peak latency values of sham-treated controls show average decreases or an increase of less than 0.04 ms. Data are mean ± s.e.m. Statistical significance of the difference between the means of control and treated groups was *p *< 0.05 in (a), and *p *< 0.01 in (b) (confidence interval = 95%; paired, one-tailed Student's *t*-test).

In contrast with the lack of effect of 5-FU treatment on DPOAEs, comparison of the changes in inter-peak latencies P2-P1 and P3-P1 with those of a sham-treated control group revealed that at the later time points of day 14 and day 56, both inter-peak latency values of 5-FU-treated animals showed marked increases (indicative of myelin damage or loss), whereas those of sham-treated controls did not (Figure 6). For example, at day 14, the P2-P1 and P3-P1 inter-peak latencies in 5-FU-treated animals increased by 0.179 ± 0.022 ms and 0.146 ± 0.050 ms, respectively, whereas in control animals these latencies decreased by 0.037 ± 0.078 ms (*p *< 0.05 compared with 5-FU group) and 0.087 ± 0.123 ms (*p *< 0.01 compared with the 5-FU group). To place these changes in context, a 0.1 ms delay in nerve impulse transmission is considered to be a highly significant functional change [104,110,111]. At day 56, the P2-P1 and P3-P1 inter-peak latencies in 5-FU-treated animals increased by 0.191 ± 0.052 ms and 0.136 ± 0.088 ms, respectively, whereas in control animals the P2-P1 inter-peak latency showed a small increase of 0.035 ± 0.075 ms (*p *< 0.05 compared with the 5-FU group), and the P3-P1 inter-peak latency decreased by 0.002 ± 0.088 ms (*p *< 0.01 compared with the 5-FU group). At earlier time points, there were no increases greater than 0.1 ms in these inter-peak latencies in either the control or the treated groups.

### 5-FU treatment causes delayed changes in expression of Olig2 and loss of myelin integrity

The results of our ABR analysis raised the possibility that 5-FU-treated animals show a syndrome of delayed white matter damage. Although our analysis of cell division and cell death following systemic treatment with 5-FU revealed a long-lasting suppression of cell division in the CC, we observed only an increased level of apoptosis in this tissue at one day after the cessation of treatment. We therefore conducted a more detailed analysis of the CC, the major myelinated tract in the rodent CNS.

**Figure 7:**
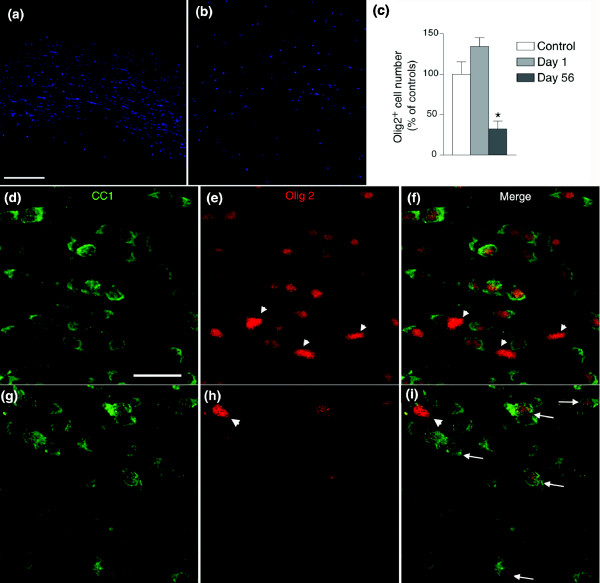
Systemic 5-FU treatment causes delayed dysregulation of Olig2 expression in oligodendrocytes in the CC. Animals were treated with 5-FU as in Figure 3 and analyzed for expression of Olig2 in the CC at various time points. There was a marked reduction in the number of such cells at 56 days (**(a) **control; **(b) **5-FU) after completion of treatment, but not at 1 or 14 days after treatment. **(c) **Percent-corrected number of Olig2^+ ^cells in the CC at day 1 and day 56 post-treatment with 5-FU, normalized to control values at each time point. Data represent averages from three animals in each group, shown as mean ± s.e.m (*p < 0.001, one-way ANOVA) in comparison with control values at each time point. The scale bar represents 150 μm. **(d-i) **Representative confocal micrographs showing loss of Olig2 expression in a subset of CC1^+ ^oligodendrocytes in the CC of a 5-FU-treated animal at day 56 in comparison with a sham-treated animal at the same time point. The reduction in numbers of Olig2^+ ^cells seen at day 56 after treatment was not associated with an equivalent fall in oligodendrocyte numbers, as determined by analysis of CC1^+ ^expression. (d-f) In control animals, there is a close equivalence between CC1 expression (d) and Olig2 expression (e); a merged image is shown in (f). Three Olig2^+ ^CC1^- ^cells can be seen in (e,f) (arrowheads), which are probably O-2A/OPCs. (g-i) In contrast, in 5-FU-treated animals there is a reduction in the number of Olig2^+ ^cells (h), but the CC of these animals contains many CC1^+ ^cells (g) that do not express Olig2 (i) (arrows, Olig2^+ ^CC1^+^; arrowheads, Olig2^+ ^CC1^- ^cells). The scale bar represents 25 μm.

Our further investigations revealed that systemic 5-FU exposure was sufficient to cause substantial delayed abnormalities in oligodendrocyte biology, in regard to both transcriptional regulation and maintenance of myelin integrity. Following treatment of six- to eight-week-old CBA mice with three injections of 5-FU (40 mg kg^-1^, every other day over 5 days), we first observed a slight increase in Olig2^+ ^cells in the CC at day 1 after completion of treatment. Examination at later time points, in contrast, revealed a substantial fall in the numbers of these cells. At day 56 after treatment, the number of Olig2^+ ^cells was markedly decreased, to 32.4 ± 9.7% (*p *< 0.001) of control levels at this time point (Figure 7a–c). Immunofluorescence staining with an anti-myelin basic protein (anti-MBP) antibody revealed that there was also markedly decreased MBP staining in animals treated with 5-FU examined 56 days after treatment (data not shown). When we double-labeled sections with the anti-CC1 antibody (to identify oligodendrocytes [112]), however, we found that the reduction in the number of Olig2^+ ^cells seen at day 56 was not matched by a similar fall in the number of CC1^+ ^ oligodendrocytes. Thus, whereas almost all CC1^+ ^oligodendrocytes in the CC of the controls were co-labeled with anti-Olig2 antibodies at day 56, in 5-FU-treated animals many CC1^+^ oligodendrocytes showed no detectable expression of Olig2 (Figure 7d–i).

**Figure 8:**
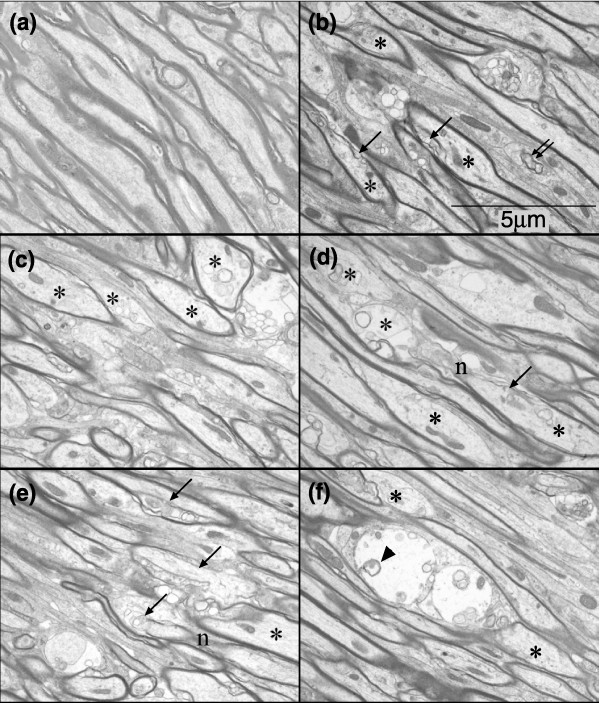
Delayed myelin and axonal degeneration in the CC caused by systemic 5-FU treatment (representative electron micrographs of longitudinal sections of axons). Sections were taken from midline coronal sections of the CC. **(a) **A representative image from a sham-treated control animal, showing normal myelinated axons and the normal axonal cytoskeleton structures; **(b-f) **representative images from a 5-FU-treated animal, showing several pathological changes of both the myelin and axonal structures. Asterisks, axonal abnormality; single arrows, damaged myelin sheaths; double arrows, myelin debris; arrowheads, engulfed myelin debris. (b) Several swollen axons with disrupted cytoskeleton (asterisks), damaged myelin sheaths (single arrows) and myelin debris (double arrows) can be seen. (c) Several swollen axons (asterisks) with or without myelin can be seen, the axoplasm of which show disruption of cytoskeleton and altered organelles. (d) Several axons (asterisks) with absent or degenerating myelin (arrows) can be seen; one axon shows a severely damaged axonal structure and myelin on one side of a node of Ranvier (n) and partially disrupted myelin sheath on the other side (arrow). (e) Several loci of myelin degeneration can be seen (arrows); one axon seems to be transected on one side of a node of Ranvier (n). An axon next to it shows partial degeneration of the myelin sheath and disruption of the cytoskeleton (asterisk). (f) Edema in what is likely to be a process of an astrocyte can be seen, with some engulfed myelin debris (arrowhead) and the adjacent axons are distorted; there are also swollen axons (asterisks) with and without myelin (arrows).

**Figure 9:**
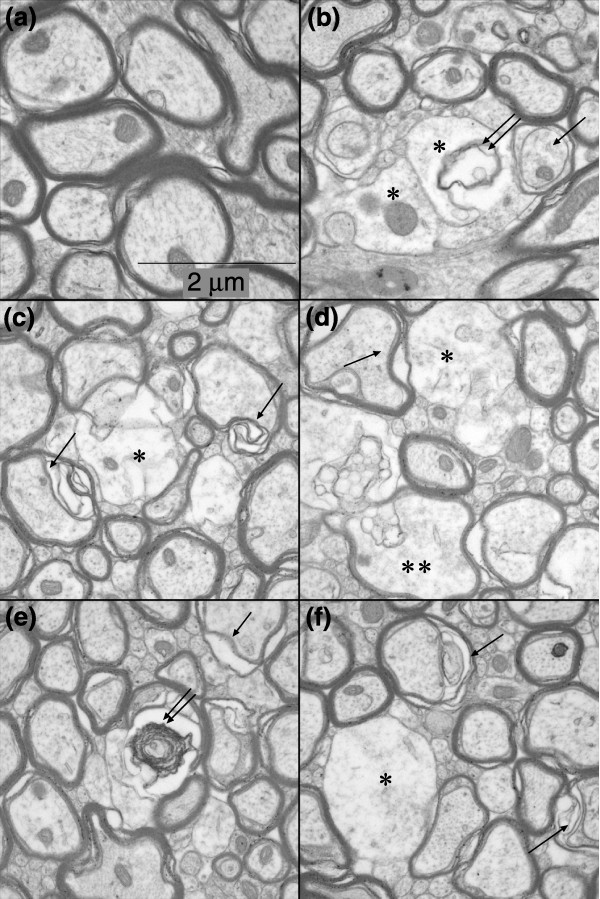
Ultrastructural evidence of myelinopathy in 5-FU-treated animals. Electron micrographs were taken from the midline transverse sections of the CC (cross-sections of the axons). **(a) **A representative image from a sham-treated control animal, showing normal myelinated axons; **(b-f) **representative images from a 5-FU-treated animal, showing multiple pathological changes of both the myelin and axonal structures. Single asterisks indicate demyelinated axons with rarefaction (that is, decreased density of the axoplasm staining possibly due to disruptions in cytoskeletal structures and organelles); double asterisks indicate an abnormal axon with partially destructed myelin sheaths; single arrows indicate inter-laminar splitting of the myelin sheaths; and double arrows indicate myelin debris. (b) Two axons with damaged myelin sheaths (asterisks), myelin debris (double arrows) and a smaller axon that seems to be detaching from its myelin sheath (single arrow) can be seen. (c) A large demyelinated axon with rarefaction of the axoplasm (asterisk) and two axons with collapsed centers and inter-laminar splitting of the myelin sheaths (arrows) can be seen, indicating on-going myelin degeneration. (d) Two large axons with completely (asterisk) or partially (double asterisks) damaged myelin can be seen, the axoplasm of which shows altered cytoskeleton and organelles. One axon has a collapsed center and inter-laminar splitting (arrow). (e) Myelin debris can be seen, possibly from a degenerating axon (double arrows) and an axon with inter-laminar splitting (arrow). (f) A demyelinated axon with rarefaction of the axoplasm and possible axonal swelling (asterisk) and two neighboring axons with inter-laminar splitting (arrows) can be seen.

Ultrastructural analysis of the CC of animals 56 days after treatment supported the interpretation of our immunocytochemical analyses that many oligodendrocytes were present at this time point, but also demonstrated the presence of abundant myelin pathology. As shown in Figure 8, midline longitudinal sections of CC displayed scattered foci of demyelinated axons, including partial or complete loss of myelin sheaths and increases in inter-laminar splitting of the myelin sheaths. Analysis of transverse sections (Figure 9) provided further evidence of myelin vacuolization and breakdown. It was also of interest to note the axonal pathology observed in these ultrastructural studies. Transverse sections revealed degenerating axons with multi-laminated structures and collapsed centers, swelling of axons and altered axonal cytoskeleton and organelles. In the transverse sections, pathological changes in axons were also readily apparent and included axonal swelling and focal degeneration of the axoplasmic cytoskeleton and microtubules.

**Figure 10:**
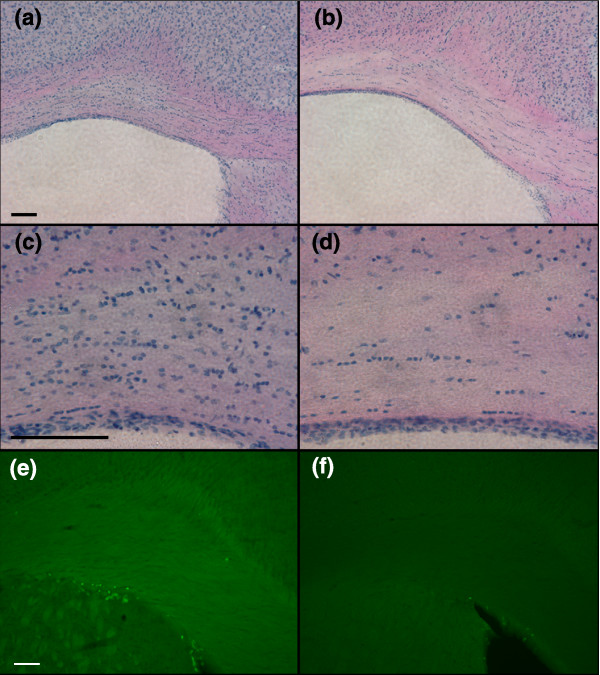
5-FU treatment causes reduced cellularity and loss of myelin basic protein (MBP) at 6 months after treatment. Representative images of hematoxylin and eosin staining from the periventricular region of **(a,c) **a control animal and **(b,d) **a 5-FU-treated animal. (c) Partial enlargement of the CC shown in (a); (d) partial enlargement of the CC shown in (b). (a) Normal cellular density is seen in the CC of the control; (b) in the CC from a 5-FU-treated animal, the cellular density in the CC has decreased markedly. **(e,f) **The expression of MBP seen in control animals (e) (the fiber-like green fluorescence staining in the CC and white-matter tracts in the peri-ventricular striatum) is greatly reduced in treated animals (f). The bright green punctuated fluorescent staining is BrdU^+ ^cells, which are present in control animals but greatly depleted in treated animals. All sections were processed at the same time and all images were taken under equal exposure times. The scale bar represents 100 μm.

Despite the presence of BrdU^+^ Olig2^+ ^cells in day 56 CC, raising the possibility of repair of the myelinopathy found at this time point, examination of animals six months after 5-FU treatment revealed eventual loss of almost all cells and myelin in this tissue. Hematoxylin and eosin staining revealed markedly decreased cellularity in the CC in treated animals at the 6 month time point, along with markedly decreased levels of MBP in the CC and in the white-matter tracts of the striatum of treated animals (Figure 10). In agreement with the majority of the cell bodies in the mature CC belonging to oligodendrocytes or glial progenitor cells, the decrease in the number of CC1^+ ^cells at 6 months matched the decrease of Olig2 labeling (data not shown), confirming loss of oligodendrocytes at this time point.

### 5-FU treatment causes only transient brain vasculature endothelial cell apoptosis and CNS inflammation in a subset of treated animals

The occurrence of delayed damage to the CNS following irradiation has been a subject of interest for many years, and both vascular damage and delayed inflammatory reactions have been implicated as being important in the adverse effects of this treatment on the CNS [113-116]. To begin to determine whether similar mechanisms might be relevant to analysis of the delayed effects of 5-FU administration, we examined microglial activation and endothelial cell apoptosis in 5-FU-treated animals.

**Figure 11:**
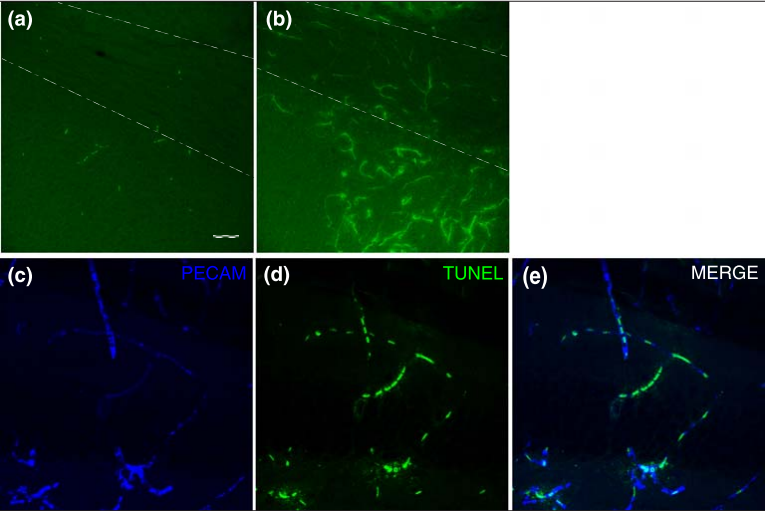
5-FU induces transient inflammation and apoptosis of microvasculature endothelial cells in a subset of treated animals. Representative photographs showing the inflammatory response on day 1 after treatment with **(a) **vehicle or **(b) **5-FU treatment as indicated by immunostaining for the activated microglia/macrophage marker F4/80. The basal level of F4/80 staining was very low in the controls but increased after treatment. These sections are from the CC (the region between the two dotted lines), with similar evidence of inflammation seen in the DG and cortex of this same mouse. The scale bar represents 50 μm. **(c-e) **TUNEL/PECAM (CD31) double immunostaining was performed in 5-FU-treated animals, with representative images taken from the DG showing double-labeling of the vascular endothelial cell marker PECAM (CD31) with TUNEL^+ ^nuclei. In the subset of animals in which evidence of endothelial cell death was observed, similar TUNEL^+ ^profiles were also found in the cortex and CC.

Unlike the consistent observations of microglial activation in the irradiated CNS [116], such evidence of inflammation following treatment with 5-FU was observed in only one of ten treated animals and only at day 1 after the cessation of treatment. Inflammatory reactions were examined in sections labeled with antibody directed against the mouse antigen F4/80, a 160 kDa glycoprotein expressed by activated murine microglia and macrophages [117]. We found that F4/80 staining was markedly increased at day 1 in one of the ten mice treated with 5-FU (Figure 11a,b). In this animal, there was diffuse microglial activation throughout the brain, including the primary motor cortex, CC, periventricular striatum and hippocampus. The activation of microglia seemed to be an acute inflammatory reaction, however, since it was not found in any treated animals at later time points. Thus, inflammation was not a frequent response to treatment with 5-FU, and no prolonged inflammatory reactions similar to those seen following irradiation were observed in our experiments.

Damage to the vasculature following irradiation has also been suggested as a possible contributor to delayed CNS damage but, as for inflammation, it seems unlikely that such damage contributed to the delayed effects of 5-FU administration. Analysis of TUNEL labeling in 5-FU-treated animals revealed a subset of animals (four out of ten total treated animals from two independent experiments) that showed markedly increased diffuse TUNEL^+^ nuclei; the distribution, morphology and size of these nuclei resembled those of the microvasculature endothelial cells of the CNS (Figure 11c–e). Double-labeling to visualize expression of the vascular endothelial cell marker PECAM/CD31 [118] confirmed that these apoptotic cells were vascular endothelial cells (Figure 11c–e). However, these indications of vascular damage were seen only in a subset of animals examined 1 day after the cessation of treatment and were not observed in animals examined at any later time points.

## Discussion

Our studies demonstrate that systemic treatment with 5-FU is associated with both acute and delayed toxicity reactions, outcomes that are of particular concern because of the use of this agent in the treatment of many cancers. As in our recent studies on cisplatin, cytarabine and carmustine [79], *in vitro *analysis of vulnerability to 5-FU revealed that lineage-restricted progenitor cells of the CNS and non-dividing oligodendrocytes were vulnerable to the effects of 5-FU at or below clinically relevant exposure levels. Thus, toxicity of 5-FU was not limited to dividing cells. Toxicity of 5-FU was not limited to induction of cell death and was also associated with suppression of O-2A/OPC division, even when applied transiently at exposure levels that represent small fractions of the CNS concentrations achieved during cancer treatment. Although previous *in vitro *studies on neurons and oligodendrocytes also observed vulnerability of these cells [119,120], the effective concentrations used in our present study are considerably lower than those used in previous studies. Our *in vitro *analyses also predicted the acute *in vivo *effects of 5-FU with considerable accuracy, just as was the case with our previous studies on cisplatin, BCNU and cytarabine [79]. 5-FU exposure transiently increased apoptosis and suppressed proliferation for extended periods of time in the SVZ, DG and CC. Cell-type-specific analyses confirmed that the main populations affected *in vivo *were also progenitor cells and oligodendrocytes. Suppression of progenitor cell proliferation was also seen *in vitro *in analyses of division and differentiation in clonal families of cells.

This study is the first to demonstrate that delayed degenerative damage can be caused by systemic application of a single chemotherapeutic agent (5-FU) and does not require the concurrent presence of cancer to manifest, as well as the first to provide an animal model of delayed damage to white matter associated with the systemic administration of chemotherapy. These results are of particular interest in the context of many clinical reports that have identified neurotoxicity as a complication of treatment regimens in which 5-FU is a component. Although most reports of 5-FU-associated neurotoxicity indicate a relatively acute onset, a delayed demyelinating cerebral complication reminiscent of multifocal leukoencephalopathy has also been increasingly reported in patients treated with chemotherapy regimens that include 5-FU [24,53-78]. Although 5-FU is used most extensively in the treatment of colorectal cancers, it is also an important component of adjuvant therapies for the treatment of a variety of other cancers, including breast [121-128], gastric [129-136], pancreatic [137-142] and lung [129,143,144], and is thus given to large numbers of patients. Neurological symptoms may occur in some patients several months after adjuvant therapy with 5-FU and include declines in mental status, ataxia and the appearance of prominent multifocal enhancing white matter lesions detectable by MRI. In addition, both acute and delayed neurological side effects have been observed for many other chemotherapeutic agents [9,14,23,26,27,29-31,33,145-154], and it will be of interest to determine whether the pattern of degenerative changes observed with 5-FU exposure is representative of delayed changes associated with other chemotherapeutic agents.

We also have provided several novel findings regarding the problem of delayed white matter damage caused by 5-FU exposure. Our findings of aberrant regulation of Olig2 expression, with the presence of many Olig2-negative oligodendrocytes at 56 days after treatment, provide the first indication that chemotherapy alters the normal expression of important transcriptional regulators in oligodendrocytes. Our ultrastructural studies demonstrate extensive myelin pathology at this time point, along with indications of neuronal pathology. It is not yet known whether damage to myelin precedes damage to neurons (as is thought to occur in multiple sclerosis (see, for example [155-162]), or whether neuronal damage occurs concurrent with or preceding myelin pathology. The vulnerability of oligodendrocytes to 5-FU *in vitro *and the increased apoptosis in these cells following 5-FU exposure *in vivo*, however, suggests strongly that oligodendrocytes are a direct target of this anti-metabolite. Although this is a somewhat surprising result (in that 5-FU has been thought to target dividing cells specifically, while oligodendrocytes do not divide in the conditions used in our experiments), previous studies have shown that experimental derivatives of 5-FU, and its metabolites, also cause myelin damage *in vitro *and *in vivo *[163,164]. Whether 5-FU derivatives such as capecitabine (an orally active form of 5-FU) cause similar damage is not yet known, but the presence of the activating enzyme for this drug (thymidine phosphorylase) in white-matter tracts [165] makes this a matter of concern.

Although the continuing presence of at least some BrdU^+ ^cells in the CC at 56 days offered the possibility that the damage to myelin occurring at this time point might be reversible, analysis at 6 months demonstrated a striking loss of cells and of MBP. Thus, it appears that even a short-term exposure to 5-FU can cause long-term and apparently irreversible damage to white-matter tracts.

Analysis of alterations in myelination caused by chemotherapy would benefit enormously from the ability to conduct functional analysis in a non-invasive manner, and our analysis of alterations in inter-peak latencies in ABRs provides a tool of particular potential interest in this regard, as well as revealing a novel form of chemotherapy-induced neurological damage. Despite extensive investigations of ototoxicity induced by exposure to cisplatin (for reviews, see [166-171]), such studies appear to have been focused exclusively on the effects of chemotherapy on hair cells and cochlear function and have not used ABR analysis of inter-peak latencies to analyze changes that may be related to white matter damage. Thus, our ABR analyses seem to provide the first demonstration of adverse effects of chemotherapy on a functional outcome related to CNS myelination. ABR inter-peak latency analysis has been used, however, to study myelination-related maturation and function of the auditory pathway in normal infants in conditions in which myelination is compromised (for example, iron deficiency, fetal cocaine syndrome) and in experimental animals [105,106,108,172-175]. Thus, this approach provides a non-invasive functional analysis of a myelination-related outcome measure that can be used in both experimental animals and human populations.

The progressive alterations in ABR inter-peak latencies observed in our studies also highlight the fact that at least some of the delayed damage associated with 5-FU administration is greater than the damage observed acutely. The ability to study progressive deterioration in the same animals over prolonged periods will make this approach of particular value in further investigations of these changes. Moreover, because of the ease of conducting such studies in humans, such analysis may provide a simple, non-invasive approach to the analysis of adverse effects on white matter complementary to the imaging-based detection of leukoencephalopathy.

The underlying causes of delayed damage induced by chemotherapy will be the subject of continued investigation, but the observations that vascular damage and inflammatory reactions were rare and were observed only at short intervals after completion of treatment makes it seem unlikely that these are causally important. This is in striking contrast to the effects of irradiation, where inflammation is thought to be essential in delayed suppression of hippocampal neurogenesis [115,116]. It is possible that the appearance of delayed damage following 5-FU treatment reflects the combined effects of delayed oligodendrocyte death and a loss of the progenitor cell populations required for replacement. Recent findings that aging is associated with a loss of expression of important transcriptional regulators, including Olig2, in oligodendrocytes [176] and may be associated with degenerative white matter changes [177-185] also raises the possibility, however, that the effect of 5-FU results from an acceleration of the normal aging processes.

Our findings also raise the question of whether multiple pathological changes contribute to the effects of chemotherapy on cognition. The ability of irradiation to the CNS to suppress the generation of new neurons in the hippocampus has been suggested to be relevant to the understanding of cognitive impairment associated with this particular form of cancer treatment [115]. Although reduced numbers of dividing hippocampal neuronal progenitors are also seen in association with exposure to 5-FU, BCNU or cytarabine [79], the additional damage to white-matter tracts caused by chemotherapy would be expected to impair normal neuronal impulse conduction (in accordance with the changes in ABR latency seen here) and thus might also contribute to alterations in cognition. It is particularly interesting in this regard that recent studies on breast cancer patients treated with adjuvant chemotherapy have revealed that, relative to controls, patients had slower speeded processing and altered fractional anisotropy (a measure of white matter integrity) in the corpus callosum. It has been suggested that these white matter changes are related to the cognitive deficits that may be associated with treatment with systemic chemotherapy [186].

As adverse effects on several normal tissues have been observed for almost all classes of chemotherapeutic agents [19-22,187] (including alkylating agents [29,30], anti-metabolites [23-26,57], methotrexate [27] and even anti-hormonal agents [31-37]) and such treatments will clearly remain the standard of care for cancer patients for many years to come, the need to understand such damage better is great. Indeed, some of the most important advances in the treatment of cancer have emerged from the study of such damage, the necessary first step in its prevention. Moreover, evaluation of potential new therapeutics that does not include adequate analysis of these potential toxicities may lead to the approval of treatments that are no better than existing treatments in avoiding serious damage to normal tissue. The clinical study of such side effects does not provide the experimental foundations required for the analysis of such problems. Indeed, treatment for neurological complications of 5-FU treatment has largely been ineffective so far, with some patients responding to immediate discontinuance of chemotherapy and steroid treatment [57,60], but with others continuing to deteriorate and, in some severe cases, progressing to death [188]. In contrast, recent studies on the toxicities *in vitro *and *in vivo *of several chemotherapeutic agents [79,189], and our discovery of an animal model for delayed damage to the CNS caused by chemotherapy, provide experimental foundations that should prove of great value in the discovery and evaluation of therapies that either allow selective killing of cancer cells or offer selective protection to the normal cells of the body.

## Materials and methods

Most materials and methods are as described in [79] and are presented here in brief.

### Preparation of primary cell cultures

*In vitro *studies were performed on purified cultures of primary CNS cells isolated from the developing rat CNS. Purified populations of neuroepithelial stem cells, neuron restricted precursor cells, glial restricted precursor cells, O-2A/OPCs, oligodendrocytes and astrocytes were all prepared and grown as described previously [79]. HUVECs (Cambrex) were cultured in endothelial growth medium (EGM-2) and used within two passages after thawing. Cancer cell lines used were established breast cancer cell lines (MCF-7 and MDA-MB-231), ovarian cancer (ES-2) cells, L1210 lymphocytic leukemia and EL-4 lymphoma cells, a meningioma cell line and two cell lines isolated from patients with glioblastoma multiforme (UT-4 and T98 cell lines); these were grown as previously described [79].

### *In vitro *toxicity and viability assay

*In vitro *toxicity studies involved microscopic analysis of staining with the 3,(4,5-dimethylthiazol-2-yl) 2,5-diphenyl-tetrazoliumbromide (MTT) assay in combination with 4',6-diamidino-2-phenylindole (DAPI) and staining with cell-type-specific antibodies, as previously described [79]. Each experiment was carried out in quadruplicate and was repeated at least twice in independent experiments. Data points represent means from single experiments and error bars shown in figures represent ± standard error of the mean (s.e.m).

### Clonal analysis

Clonal analysis of O-2A/OPC division and differentiation was carried out as described previously [92-94]. One day after plating, cells were exposed for 24 h to low-dose 5-FU (0.01 μM), a concentration that did not cause significant killing of O-2A/OPCs in mass culture. The number of undifferentiated progenitors and differentiated oligodendrocytes was determined in each individual clone from a total of 100 clones in each condition by morphological examination and by immunostaining to confirm cell-type identification. Experiments were performed in triplicate in at least two independent experiments.

### Chemotherapy application *in vivo*

For *in vivo *experiments, 6–8-week-old CBA mice were treated with chemotherapy under approved protocols. 5-FU (Sigma) was administered by i.p. injections. Animals received 5-FU as three consecutive injections every other day (40 mg kg^-1 ^body weight). Control animals received equal amounts of 0.9% NaCl i.p. Animals were sacrificed on days 1, 7, 14 and 56 and 6 months after completion of treatment with 5-FU (where day 0 is the time of the last injection of the agent). For all *in vivo *experiments, animals were perfused transcardially with 4% paraformaldehyde in phosphate buffer (pH 7.4), under deep anesthesia using Avertin (tribromoethanol; Sigma; 250 mg kg^-1^, 1.2% solution).

We chose the *in vivo *dosage on the basis of conversion from human treatment dosage to an equivalent mouse dosage and previous animal studies of 5-FU effects in mice. As is standard practice, we used a conversion factor of 3 [190-194] to calculate the equivalent mouse dose range (20–1,167 mg kg^-1^) from the clinical human treatment dose range (60–3,500 mg m^-2^). On the basis of animal studies in mice (for example, [195-197]), in which doses of 5-FU used ranged from 40–200 mg kg^-1^, we first used 60 mg kg^-1 ^every other day for three doses as the initial trial treatment. As this treatment caused death in half of treated CBA mice one week after completion of treatment, we lowered the dosage to 40 mg kg^-1^, which was tolerated well by the animals (it caused less than a 10% increase in death over a six-month period compared with sham-treated controls). The differences in tolerance to 5-FU treatment between our study and others may result from the different mouse strains used. In clinical practice, patients are often given the highest tolerated dosage of chemotherapy to achieve maximum fractional kill of the malignant cells. Considering this situation, we determined the appropriate *in vivo *dosage to be 40 mg kg^-1^.

### Immunofluorescence, TUNEL staining and analysis of BrdU incorporation *in vivo*

All *in vivo *analysis was carried out as described in [79]. Using free-floating sections (40 μm), detection of nuclear profiles with DNA fragmentation, a hallmark of apoptosis, was performed using a TUNEL assay combined with DAPI counterstaining to visualize nuclear profiles. To combine TUNEL staining with immunofluorescence staining for different cell lineage markers, TUNEL staining was performed first, followed by labeling with one of the following primary antibodies for 24–48 h: mouse anti-NeuN (1:500, Chemicon), goat anti-DCX (doublecortin; 1:500, Santa Cruz), rat anti-S-100β (1:2,500, Swant), mouse anti-MBP (1:1,000, Chemicon), mouse anti-GFAP (1:2,500, DAKO), rabbit anti-Olig2 (1:1,000, a gift of David Rowitch), mouse anti-CC1 (1:300, Calbiochem; which was used under conditions [101] in which specificity for oligodendrocytes is preserved and no double-labeling with GFAP^+ ^astrocytes was observed), rat anti-CD31/PECAM (1:500, Chemicon) and rat anti-F4/80 (Abcam). All secondary antibodies, generated in donkey (anti-rat, anti-rabbit, anti-goat and anti-mouse), were coupled to TritC, FitC or Cy5 (Jackson ImmunoResearch) for *in vivo *staining and were used according to the species of primary antibody. Fluorescent signals were detected using a confocal laser scanning microscope Leica TCS SP2 and a 40× oil immersion lens, with pinhole settings corresponding to an optical thickness of less than 2 μm used to avoid false positive signals from adjacent cells.

To label the proportion of dividing cells engaged in DNA synthesis *in vivo*, mice received a single injection of BrdU (50 mg kg^-1^ body weight, dissolved in 0.9% NaCl) given i.p. 4 h before perfusion. Anti-BrdU antibody was used to identify BrdU^+ ^ cells by standard techniques (as in [79]). A minimum of 50 BrdU^+ ^ cells was counted for each labeling condition in each animal (n = 3 animals in each group examined), with the sole exception of the DG of the animals examined 56 days after cytarabine treatment, for which an identical number of sections were examined as in controls, but the frequency of labeled cells was not sufficient to reveal 50 cells in these sections. Quantification of BrdU^+^ cells was accomplished with unbiased counting methods. BrdU immunoreactive nuclei were counted in one focal plane to avoid over-sampling. Brain structures were sampled either by selecting predetermined areas on each section (lateral SVZ) or by analyzing the entire structure on each section (CC and DG). Differences were considered significant when *p *< 0.01.

#### SVZ

BrdU^+ ^cells were counted in every sixth section (40 μm) from a coronal series between interaural anterior-posterior (AP) +5.2 mm and AP +3.9 mm (the anterior commissure crossing). BrdU^+ ^cells were counted along the lateral ventricular wall up to 200 μm distance from the lateral ventricle wall.

#### CC

BrdU^+ ^cells were counted in every sixth section (40 μm) from a coronal series between interaural AP +5.2 mm and AP +3.0 mm in the entire extension of the rostral and medial part of the CC and analyzed as for the SVZ.

#### DG

BrdU^+ ^cells were counted in every sixth section (40 μm) from a coronal series between interaural AP +2.5 mm and AP +1.1 mm. BrdU^+ ^cells were counted in the area of the dentate gyrus, including the hilus, subgranular zone and the granule cell layer and analyzed as for the SVZ. Quantitative data in all figures are presented as mean percentage normalized to control animals. Error bars represent ± s.e.m.

To analyze BrdU incorporation in specific cell types, anti-BrdU immunostaining was combined with immunolabeling to identify DCX^+^ neuronal precursor cells [95], Olig2^+ ^oligodendrocyte precursor cells (defined as cells that were BrdU^+ ^and Olig2^+^, in order to discriminate these cells from Olig2^+ ^non-dividing oligodendrocytes [97,98,101]) and GFAP^+ ^cells; the latter would have been astrocytes in the CC or DG or, in the SVZ, may also have been stem cells [96]. Labeling and confocal analysis was carried out as for the combination of immunolabeling with TUNEL staining.

### Auditory brain stem responses

Baseline ABRs were measured in each animal one day before initiation of treatment. After treatment ended, follow-up ABR tests were conducted on each animal at various points during a time course of 56 days. For the measurement, mice were anesthetized with xylazine (20 mg kg^-1 ^i.p.) and ketamine (100 mg kg^-1 ^i.p.) [198]. Needle electrodes were inserted at the vertex and pinna of the ear, with a ground near the tail. ABR potentials were evoked with click stimuli at 80 dB SPL. The response was amplified (10,000×) and 1,024 responses were averaged with an analog-digital board in a LabVIEW-driven data-acquisition system. For comparison of change of latencies, the wave peaks P1, P2 and P3 were identified by visual inspection at recorded wave forms, and the inter-peak latencies of wave P2-P1 and P3-P1 computed. The change of inter-peak latencies was calculated as L_t _– L_0 _(where L_t _is the inter-peak latency values at day 1, day 7, day 14, or day 56 post-treatment; and L_0 _is the baseline inter-peak latency values one day before treatment initiation).

### Images and data processing and statistics

Digital images were captured using a Nikon Eclipse E400 upright microscope with a spot camera (Diagnostic Instruments) and the spot advanced software for Macintosh (Diagnostic Instruments), or using the confocal laser scanning microscope (Leica TCS SP2). Paired or unpaired Student t-tests were used for statistical analyses where applicable.

### Electron microscopy

The animals were anesthetized and injected with heparin and perfused with a 0.1 M phosphate buffered 4.0% paraformaldehyde/2.5% glutaraldehyde fixative. The brain was removed and allowed to fix overnight at 4°C and the CC was then sectioned sagitally and coronally at approximately 1.0 mm. The sections were rinsed in 0.1 M sodium phosphate buffer, post-fixed in buffered 1.0% osmium tetroxide, dehydrated in a graded series of ethanol to 100%, transitioned to propylene oxide and infiltrated with EPON/Araldite epoxy resin overnight. They were embedded into mold capsules (BEEM) and polymerized for 2 days at 70°C. Sections of 1 μm were cut on to glass slides and stained with toluidine blue to determine areas to be thin sectioned at 70 nm onto grids. The grids were stained sequentially with uranyl acetate and lead citrate. A Hitachi 7100 transmission electron microscope was used to examine and digitally capture images using a MegaView III digital camera (Soft Imaging).

## Additional data files

Additional data file 1 shows a representative confocal micrograph of TUNEL^+ ^cells co-labeled with cell-type-specific markers. (A-A") show a TUNEL^+ ^ DCX^+ ^ cell in the sub-ventricular zone; (B-B") show a TUNEL^+^ Olig2^+ ^cell in the CC; (C-C") show a TUNEL^+ ^GFAP^+ ^cell in the CC. The scale bars represent 10 μm.

## Supplementary Material

Additional data file 1 A representative confocal micrograph of TUNEL^+ ^cells co-labeled with cell-type-specific markers. (A-A") show a TUNEL^+ ^DCX^+ ^cell in the sub-ventricular zone; (B-B") show a TUNEL^+^ Olig2^+ ^cell in the CC; (C-C") show a TUNEL^+ ^GFAP^+ ^cell in the CC. The scale bars represent 10 μm Click here for additional data file
